# Neonatal and maternal adverse outcomes among low-risk nulliparous women compared with multiparous women at 37–41 weeks of gestation: a cohort study in South China

**DOI:** 10.3389/fmed.2025.1691707

**Published:** 2026-01-05

**Authors:** Yiping Liu, Bo Li, Jie Liu, Bin Wen, Chuan Xiao, Yaru Chen, Fanjuan Kong

**Affiliations:** 1 NHC Key Laboratory of Birth Defect for Research and Prevention (Hunan Provincial Maternal and Child Health Care Hospital), Changsha, China; 2 Hunan Provincial Institute of Schistosomiasis Control and Prevention (The Third People's Hospital of Hunan Province), Yueyang, Hunan, China

**Keywords:** 37–41 weeks of gestation, low-risk, multiparous women, neonatal and maternal adverse outcomes, nulliparous women

## Abstract

**Objective:**

The objective of this study was to compare the differences in neonatal and maternal adverse outcomes between low-risk full-term nulliparous (women giving birth for the first time) and low-risk full-term multiparous (women who have given birth previously) using a database from 18 hospitals.

**Methods:**

The cohort study, conducting from January 2019 to December 2019, described the frequency of maternal and neonatal adverse outcomes in low-risk nulliparous women and multiparous women who labored at 37–41 weeks of gestation. The association between maternal parity and the risk of neonatal and maternal adverse outcomes were analyzed based on multivariate Poisson regression.

**Results:**

Of the 75,033 live births during the study period, 49,935 (66.55%) met the inclusion criteria, including 44.18% of nulliparous women and 55.82% of multiparous women. After adjustment, the risk of maternal adverse outcomes was 0.56 times reduced in multiparous women compared to nulliparous women (aRR 0.56, 95% CI 0.52–0.65, 44% lower risk), and the incidence of complication interventions in multipara was lower than in nullipara (aRR 0.76, 95% CI 0.59–0.99). Macrosomia and low birth weight were the most common neonatal adverse outcomes in the two groups. There was no statistically significant difference in neonatal adverse outcomes between multipara and nullipara after controlling for confounders.

**Conclusion:**

Among low-risk women who delivered at 37–41 weeks of gestation, the risk of maternal adverse outcomes and the incidence of complication interventions in nulliparous women were higher than in multiparous women. There was no statistically significant difference in adverse neonatal outcomes between two groups after adjustment.

## 1 Introduction

Influenced by factors such as social development and the elevated education among women, the number of older first-time mothers has been rising (1). In addition, due to the adjustment of Chinese fertility policy and the liberalization of the “second-child policy,” the proportion of advanced maternal age (>35) has increased significantly in China (2), especially advanced age among multipara (3). Previous studies have demonstrated that increasing age will lead to a decline in maternal physical function, as well as the risk of pregnancy complications and adverse pregnancy outcomes during pregnancy are also significantly higher than that of age-appropriate pregnant women (4–7). Therefore, the survey of the pregnancy characteristics and pregnancy outcomes of nullipara and multipara after the Chinese second-child policy is essential to take preventive measures in advance and carry out crucial interventions to prevent or reduce the occurrence of adverse pregnancy outcomes.

There were 12 million births in 2020 in China (8), most of which occurred at 37–41 weeks of gestation. Currently, numerous researches only focused on the association between maternal age at delivery and pregnancy outcomes (9, 10). To our knowledge there is no relevant report on whether maternal complications and pregnancy outcomes are consistent among full-term and low-risk women at 37–41 weeks of gestation in China. Therefore, we think our study is the first to investigate the differences in the distribution of pregnancy risk factors and adverse pregnancy outcomes between nulliparous women and multiparous women who labored at 37–41 weeks of gestation in China, in order to provide data to support the reduction of the incidence of adverse pregnancy outcomes and the promotion of maternal and child health.

## 2 Materials and methods

### 2.1 The study population

This study was a population-based cohort study. From January 2019 to December 2019, pregnant women who gave birth in 18 monitoring hospitals in Hunan Province were selected as the study population. The study included pregnant women who met the inclusion criteria. Inclusion criteria for participation included: (i) 37–41 weeks of gestation; (ii) singleton; (iii) no gestational hypertension; (iv) no gestational diabetes. Participants were excluded when their information was incomplete. All pregnant women who participated in our cohort study were divided into nullipara and multipara. Nullipara was defined as a woman with no previous delivery (zero delivery) and multipara was defined as a woman with at least one prior delivery (one or more times deliveries).

### 2.2 Information collection

A specific maternal and child questionnaire was used to collect the following information about age, marriage, education, mode of delivery, number of pregnancies, parity, place of delivery, previous cesarean sections, medical treatment, gestational age and fetal presentation. Information about maternal adverse complications included uterine rupture, placenta previa, placental abruption, uterine atony, post-partum hemorrhage, infections, anemia, tear of soft birth canal and retention of placenta. Information about neonatal adverse outcomes included intrapartum stillbirth, antepartum stillbirth, preterm birth, Apgar score, low birth weight, macrosomia. Based on the maternity examination archives and delivery medical records of the research population in the institution, the information was filled out by uniformly trained staff of maternal and child healthcare institutions. Meanwhile, the information was entered into the Hunan Provincial Population Health Information Platform in real time through direct reporting on the Internet. Our research did not contain direct personal information identifiers and was approved by the Medical Ethics Committee of Hunan Maternal and Child Health Hospital (2020S072).

### 2.3 Outcomes metrics definition

In this study, the neonatal and maternal adverse outcomes were defined as follows (11). The neonatal adverse outcomes of interest were intrapartum stillbirth, antepartum stillbirth, preterm birth, Apgar score, low birth weight, macrosomia. Stillbirth was defined as the death of the fetus in the uterus after 20 weeks of gestation, divided into antepartum stillbirth (the death of a fetus before delivery) and intrapartum death (the death of a fetus in childbirth or during childbirth). Preterm birth was defined as delivery before 37 weeks of gestation. The Apgar score comprised color, heart rate, reflexes, muscle tone, respiration and was reported at 1 and 5 min after birth for all infants. Low birth weight was defined as birth weight <2,500 g. Macrosomia was defined as birth weight >4,000 g. Maternal adverse outcomes were defined as diseases occurring during the perinatal period (after 28 weeks of gestation to 1 week postpartum) that can endanger maternal health. Uterine rupture was defined as a rupture of the uterine body or lower segment of the uterus during delivery or at the end of pregnancy. Placenta previa was defined as the attachment of the placenta to the lower segment of the uterus after 28 weeks of gestation, with the lower edge of the placenta reaching or covering the inner opening of the cervix. Placental abruption was defined as the premature detachment of the placenta from the uterine wall partially or completely, before birth and after 20 weeks of gestation. Uterine atony was defined as inadequate contraction of the uterus during labor and after delivery. Post-partum hemorrhage was defined as a blood loss of more than 500 mL for vaginal delivery and more than 1,000 mL for cesarean delivery within 24 h of giving birth. Infections included abortion-related infections, puerperal infections, uterine incision infections, urinary tract infections, upper respiratory tract infections, thrombophlebitis, and other systemic infections/sepsis. Anemia in pregnancy was defined as hemoglobin < 110 g/L. Tear of soft birth canal was defined as lacerations or ruptures in the soft birth canal (vagina, cervix, perineum, or labia) during childbirth. Retention of the placenta was defined as the failure of the placenta to detach and expel from the uterus within 30 min after childbirth.

### 2.4 Data accuracy and control mechanism

The data for this cohort study were derived from Maternal and Child Health Information Direct Reporting Management System. Data were collected by designated obstetricians using standardized case-report forms for every admitted pregnant woman at each participating hospital. The completed forms underwent the structured multi-tiered audits. They were first reviewed and consolidated at the hospital level monthly. Subsequently, these forms were submitted to district, municipal, and provincial maternal and child health centers for further verification against medical records before being entered into the national database. To ensure the high quality and accuracy of the data, a rigorous multi-level quality control mechanism was implemented throughout the process. Higher-level agencies performed scheduled quality control reviews annually, which involved re-abstracting a random sample of cases to check for discrepancies.

### 2.5 Statistical analysis

Categorical variables were presented as rates or percentages. The Chi-square Test was used to compare the differences in demographic features and delivery information between two groups. Multivariate Poisson regression was utilized to assess the association of parity (nullipara as reference) with the risk of neonatal and maternal adverse outcomes adjusting for maternal age, marital status, number of pregnancies, mode of delivery and previous cesarean sections. Results were shown as adjusted relative risks (aRR) and 95% confidence intervals (CI). All analyses were performed using SPSS 26.0 and Microsoft Excel 2010. All tests were two-tailed, with *p* < 0.05 set as the statistically significant difference.

## 3 Results

### 3.1 General characteristics

**Figure 1:**
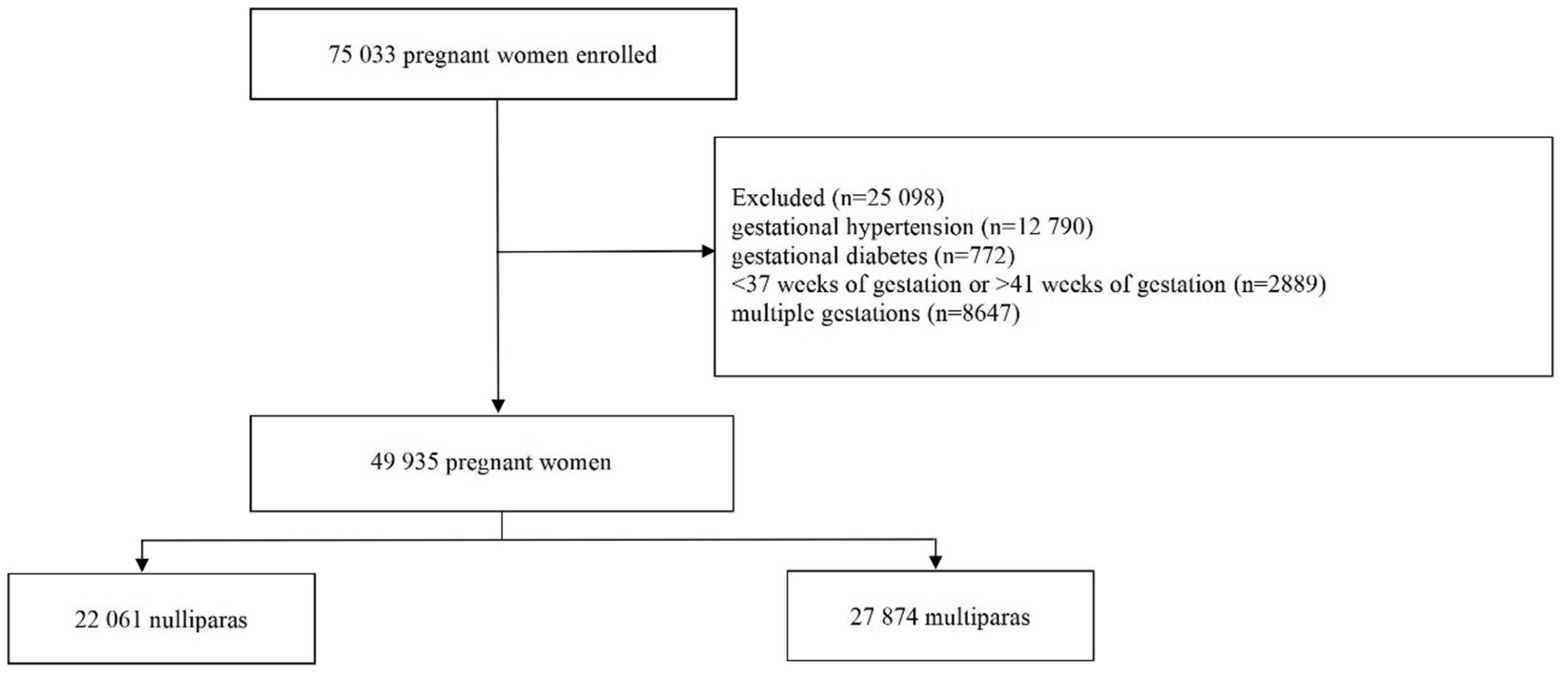
Flow diagram of the study.

Based on inclusion criteria, a total of 49,935 women were included into this research (Figure 1). Of these, 22,061 pregnant women were nulliparas (44.18%) and 27,874 pregnant women were multiparas (55.82%). 32.2% of multipara had advanced maternal age and 36.3% of multipara had previous cesarean section. Significant differences were found in nullipara and multipara for age, marital status, education, number of pregnancies and previous cesarean section (Table 1).

**Table 1:** Comparison of general characteristics between nullipara and multipara.

Characteristic	Nullipara (*n* = 22,061, %)	Multipara (*n* = 27,873, %)	*P*
Age			<0.001
<35	20,234(92.1)	18,813(67.8)	
≥35	1744(7.9)	8,932(32.2)	
Marital status			<0.001
Single/divorced/widowed/other	245(1.1)	139(0.5)	
Married	21,481(97.4)	27,607(99.0)	
Cohabitation	335(1.5)	126(0.5)	
Education			<0.001
College and above	11,860(53.8)	9,872(35.4)	
Senior middle school	7,558(34.3)	11,905(42.7)	
Junior high school	2,530(11.5)	5,877(21.1)	
Primary schools	59(0.3)	153(0.5)	
Illiteracy	49(0.2)	58(0.2)	
Number of pregnancies			<0.001
1	15,235(69.1)	0(0)	
2–3	6,196(28.1)	19,173(68.8)	
4–5	542 (2.4)	7,376 (26.5)	
>5	88(0.4)	1,289(4.6)	
Previous cesarean sections			<0.001
0	22,060(100)	17,754(63.7)	
1	0	9,168(32.9)	
>2	0	949(3.4)	

### 3.2 Comparison of delivery characteristics

64.6% of nullipara gave birth at 39–40 weeks of gestation, higher than 59.8% of multipara. 60.6% of nullipara labored in provincial (prefectures) hospitals, higher than the 41.7% of multipara. The rate of cesarean section for nullipara was 30.5%, lower than that for multipara (42.6%). The proportion of nullipara with head presentation was 97.2%, lower than of multipara (98.2%). There were significant differences in gestational age, place of delivery, mode of delivery, medical treatment and fetal presentation between nullipara and multipara (Table 2).

**Table 2:** Comparison of delivery characteristics between nullipara and multipara.

Characteristic	Nullipara (*n* = 22,061, %)	Multipara (*n* = 27,873, %)	*P*
Gestational age			<0.001
37–38 weeks	5,481(24.8)	9,940(35.7)	
39–40 weeks	14,254(64.6)	16,660(59.8)	
41 weeks	2,326(10.5)	1,272(4.6)	
Place of delivery			<0.001
Provincial (prefectural) hospital	13,370(60.6)	11,630(41.7)	
County (district) hospital	8,688(39.4)	16,202(58.1)	
At home	0(0.0)	16(0.1)	
On the way	0(0.0)	18(0.1)	
Mode of delivery			<0.001
Vaginal delivery	15,323(69.5)	15,990(57.4)	
Cesarean section	6,731(30.5)	11,870(42.6)	
Medical treatment			0.002
General anesthesia	86(0.4)	96(0.3)	
Intraspinal anesthesia/analgesia	11,501(52.1)	15,085(54.1)	
Uterine contraction drugs	11,325(51.3)	13,806(49.5)	
Antibiotic	6,134(27.8)	10,894(39.1)	
Fetal presentation			<0.001
Head presentation	21,449(97.2)	27,359(98.2)	
Breech presentation	579(2.6)	468(1.7)	
Shoulder presentation	18(0.1)	24(0.1)	
Mixed presentation	15(0.1)	21(0.1)	

### 3.3 Maternal adverse outcomes and complication interventions

The number of women with adverse maternal outcomes in nullipara and multipara were 15,612(70.8%) and 17,566(63.0%), respectively. Anemia and uterine atony were the most common maternal adverse outcomes in the two groups. The risk of maternal adverse outcomes was 0.56 times lower in multiparous women than in nulliparous women (aRR 0.56, 95% CI 0.52–0.65, 44% lower risk) after adjusting for age, marital status, number of pregnancies, mode of delivery, and previous cesarean sections. The incidence of uterine atony, tear of soft birth canal and postpartum hemorrhage were significantly more common in nullipara. Therapeutic intravenous antibiotics for infection was the most common complication intervention in the two groups. The incidence of complication interventions in multipara was lower than in nullipara (aRR 0.76, 95% CI 0.59–0.99) after adjustment for confounders (Table 3). Blood transfusion or blood products and therapeutic intravenous antibiotics were the more common complication interventions in nullipara.

**Table 3:** Comparison of maternal adverse outcomes and complication interventions between nullipara and multipara.

Characteristic	Nullipara (*n* = 22,061)	Multipara (*n* = 27,873)	Unadjusted *RR* (95%CI)	Adjusted *RR*^a^ (95%CI)
Maternal adverse outcomes	15,612(70.8%)	17,566(63.0%)	0.67(0.64,0.71) *	0.56(0.52,0.65) *
Anemia ^b^	6,572(29.8%)	8,798(31.6%)		
Intrapartum Complications ^c^				
Placenta previa	79(0.4%)	174(0.6%)		
Placental abruption	71(0.3%)	71(0.3%)		
Uterine rupture	1(0.0%)	19(0.1%)		
Tear of soft birth canal	595(2.7%)	584(2.1%)		
Postpartum Complications ^d^				
Uterine atony	1,033(4.7%)	839(3.0%)		
Postpartum hemorrhage	253(1.2%)	294(1.1%)		
Retention of placenta	124(0.6%)	156(0.6%)		
Infection #	759(3.4%)	1,076(3.9%)		
Complication interventions	614(2.8%)	709(2.5%)	0.91(0.82, 1.02)	0.76(0.59, 0.99) *
Blood transfusion or blood products	404(1.8%)	225(0.8%)		
Therapeutic intravenous antibiotics	128(0.8%)	110(0.6%)		
Other surgical treatment	85(0.3%)	57(0.5%)		
Uterine tamponade	49(0.3%)	52(0.3%)		
Massive transfusion †	25(0.1%)	35(0.1%)		
ICU	8(0.03%)	9(0.03%)		
Hysterectomy	0(0.0%)	5(0.01%)		

^a^Adjusted for age, marital status, number of pregnancies, mode of delivery and previous cesarean sections. ^b^Anemia referred to gestational anemia diagnosed during pregnancy and was not analyzed as an intrapartum or postpartum complication. ^c^ Intrapartum complications included placenta previa, placental abruption, uterine rupture, and tear of the soft birth canal. ^d^ Postpartum complications included uterine atony, postpartum hemorrhage, retention of placenta, and infection. # Infections include abortion-related infections, puerperal infections, uterine incision infections, urinary tract infections, upper respiratory tract infections, thrombophlebitis, and other systemic infections/sepsis. † Mass transfusion: whole blood ≥1,000 ml or red blood cell suspension ≥5 units. *p < 0.05. Multinomial Logit models were used for statistical analysis, nulliparous women as reference.

### 3.4 Neonatal adverse outcomes

Macrosomia and low birth weight were the most common neonatal adverse outcomes in the two groups. The risk of neonatal adverse outcomes in multipara was lower than in nullipara (RR 0.88, 95% CI 0.81–0.95). However, there was no statistically significant difference in neonatal adverse outcomes between multipara and nullipara after controlling for confounders (*p* > 0.05) (Table 4).

**Table 4:** Comparison of neonatal adverse outcomes between nullipara and multipara.

Characteristic	Nullipara (*n* = 22,061, %)	Multipara (*n* = 27,873, %)	Unadjusted *RR* (95%CI)	Adjusted *RR*^a^ (95%CI)
Neonatal adverse outcomes	1,046(4.7%)	1,494(5.4%)	0.88(0.81, 0.95)*	0.91(0.75, 1.10)
Intrapartum stillbirth	4(0.0%)	4(0.0%)		
Antepartum stillbirth	9(0.0%)	20(0.1%)		
Apgar score below 5 at 1 min	52(0.2%)	48(0.2%)		
Apgar score below 5 at 5 min	14(0.1%)	29(0.1%)		
Low birth weight: below 2,500 g	321(1.5%)	247(0.9%)		
Macrosomia: above 4,000 g	682(3.1%)	1,212(4.3%)		

^a^Adjusted for age, marital status, number of pregnancies, mode of delivery and previous cesarean sections. *p < 0.05. Multinomial Logit models were used for statistical analysis, nulliparous women as reference.

## 4 Discussion

We conducted a cohort study using information on pregnant women who labored at 37–41 weeks of gestation from 18 hospitals in Hunan Province. After adjusting for confounding factors, the risk of maternal adverse outcomes and the incidence of complication interventions in nulliparous women was higher than in multiparous women which was different from the results of other studies (12, 13). Possible reasons were as follows: Firstly, pregnant women with gestational diabetes or gestational hypertension or twin pregnancies were excluded from the study. All participants were low-risk pregnant women. Secondly, this study included women who gave birth at 37–41 weeks which was different from other research that included subjects delivering throughout all gestation periods. A 44% reduction in the risk of maternal adverse outcomes was observed in multiparous women compared with nulliparous women. From a clinical perspective, this substantial risk reduction underscored that multiparity was a strong protective factor against maternal complications. This finding highlighted the importance of tailoring patient counseling and clinical resource allocation based on parity. While multiparous women might have required less intensive monitoring for certain maternal complications, it reaffirmed the need for vigilant and supportive care for nulliparous women, who constituted a higher-risk group.

Association between parity and the risk of neonatal adverse outcomes was not observed in our study which was consistent with the findings of other studies (13). This may be partially attributed to the strengthening of prenatal care and pregnancy monitoring for pregnant women in China over the years, especially the strengthening of perinatal health care for pregnant women from the migrant population (14).

Although the incidence of adverse pregnancy outcomes and complications was low, there were around 12 million births per year in China (8). This signified that even though an event occurred rarely, its total burden can be high in terms of absolute numbers. In terms of the number of births, the proportion of “second children” in the Chinese birth population has increased significantly. It rose from about 30% in 2013 to about 50% in 2017. Although it declined since then, it was still above 40% (8). The present study showed that 66.55% of low-risk mothers delivered at 37–41 weeks of gestation, including 44.18% of nulliparous women and 55.82% of multiparous women, which was similar to the proportion of literature with the same research conditions (15).

In this study, the proportion of advanced age (age≥35) was higher among the multiparas than among the nulliparas. The risk of pregnancy complications and adverse pregnancy outcomes was significantly increased in pregnant women with advanced age compared to age-appropriate women (16). Whereas, a cohort study based in the UK found that there was no difference in postpartum massive hemorrhage, cesarean section and preterm birth between advanced age and age-appropriate women after adjusting for confounding factors (17). de Weger et al. (18) also demonstrated that maternal age was not associated with some neonatal outcomes such as preterm birth. In our research, the complications were higher in nulliparous women than in multiparous women which suggested that the management and monitoring of pregnancy should take into account not only parity and age of delivery, but also the week of gestation, so as to carry out targeted perinatal health care and intrapartum monitoring.

In China, the maternal health management services provided systematic health care for women from pregnancy to 42 days postpartum, including the establishment of a pregnancy care manual, pre-pregnancy health care, prenatal check-ups, postpartum visits. These services were beneficial for medical institutions to monitor the physical condition of all pregnant women, and assisted nullipara in a smooth delivery. In addition, the “Five-Color Management” was implemented in our country, which classified pregnant women into five colors level—green, yellow, orange, red, and purple—according to their basic conditions and pregnancy complications (19). The associated risk increased progressively from green to purple. While the WHO provided a broad framework for risk assessment and recommended a continuum of care throughout the life course, the Chinese model offered a specific, color-coded algorithmic approach for antenatal management. The “Five-Color Management” explicated management protocols for each level—ensured that interventions were precisely matched to the level of risk, from routine care to intensive, multi-specialist management in designated critical care centers. This structured response aligned with the WHO’s emphasis on improving service delivery and health systems, demonstrating one method for its practical implementation.

Our study found that the proportion of cesarean section was higher among the multiparas than among the nulliparas. Whereas, a retrospective cohort study in Hail, Saudi Arabia showed that there was no significant difference in the mode of delivery between the study parity cohort groups (20). The different research results may be explained by the fact that multiparous women were older and affected by previous cesarean sections in our study. Specifically, 36.3% of multiparous women were with previous cesarean sections. Although this variable was included as a covariate in our multivariate models, its high prevalence likely acted as a significant effect modifier. The markedly higher proportion of cesarean sections among multiparas was almost driven by previous cesarean sections. Furthermore, the inherent risk associated with a uterine scar such as elevated risks for uterine rupture and abnormal placentation, meant that the inclusion of these women influenced the overall complication landscape for the multiparous group (21). Consequently, the protected effect of multiparous women observed in our maternal adverse outcome most likely accurately reflected the experience of multiparous women without previous cesarean sections. This finding underscored a critical heterogeneity within the multiparas and highlighted that the previous cesarean sections were a key factor that could redefine clinical trajectories, meriting explicit consideration in future research and risk stratification.

Anemia was the most common maternal adverse outcome in the present study, with prevalence rates of 29.8 and 31.6% in nullipara and multipara, respectively. In a cohort study of 18,948,443 pregnant women, the severity of anemia during pregnancy was found to be associated with an increased risk of placental abruption, preterm delivery, severe postpartum hemorrhage, and fetal malformations (22). 40.05% of pregnant women globally suffered from anemia during pregnancy in 2016, with the highest prevalence (48.15%) in South East Asia (23). The absence of a significant difference by parity in our study suggested that anemia was not solely related to maternal obstetric history, but was a pervasive issue affecting all pregnant women. Therefore, our findings indicated that strategies for anemia prevention and management should be universally applied across all antenatal care programs. However, the underlying caused might differ. For instance, nulliparous women might have been more susceptible to anemia due to dietary factors (24, 25), while multiparous women could have been at risk because of depleted iron stores from closely spaced pregnancies (26).

A higher incidence of macrosomia was observed in multiparous women compared with nulliparous women. This increased incidence could be attributed to several factors. Firstly, multiparous women often had a history of previous pregnancies, which could lead to physiological adaptations that might facilitate larger fetal growth (27). Additionally, multiparous women tended to have a higher pre-pregnancy body mass index (BMI) and greater gestational weight gain, both of which were associated with higher birth weights (28, 29). Furthermore, hormonal changes and uterine capacity in multiparous women could also contribute to an environment that supported macrosomia. Collectively, these factors raised the likelihood of delivering larger infants in multiparous pregnancies.

There were some differences between this research and previous studies of low-risk pregnant women. Firstly, we focused on full-term deliveries at 37–41 weeks of gestation to provide clinicians with a reference for women delivering in this gestational age range. Secondly, previous literature reported maternal and neonatal adverse outcomes either in nullipara (30–32) or multipara (33, 34), without directly comparing outcomes based on maternal parity at delivery. Thirdly, differences in complications between nulliparous women and multiparous women remained after adjusting for age, marital status, number of pregnancies, mode of delivery and previous cesarean sections.

This study had several limitations. The absence of data on maternal weight and body mass index (BMI) before and during pregnancy, gestational weight gain, socioeconomic status, history of diseases, history of adverse pregnancy outcomes and clinical treatment constituted a potential threat to the internal validity of our study, primarily through the mechanism of residual confounding. The data of BMI was critical, as pre-pregnancy BMI and gestational weight gain were independent risk factors for complications such as macrosomia, cesarean delivery (35, 36). Thus, the observed association between multipara and macrosomia could be substantially confounded if multiparous women had a higher average BMI. Socioeconomic status was a known determinant of health-seeking behaviors, nutrition, and obesity, which can influence pregnancy outcomes (37). Besides, because of the limited sample size in this study, we did not assess the association of parity with uncommon outcomes such as maternal mortality. In addition, our study compared mode of delivery between nullipara and multipara which was regarded as baseline characteristics data without adjusting or stratifying for previous cesarean, which cannot better reveal the association between parity and cesarean section. Furthermore, the definition of “low-risk” might not have encompass all potential high-risk conditions, such as maternal obesity and so on. The inclusion of women with such unmeasured conditions could have introduced residual confounding. Therefore, future studies would benefit from adopting a more comprehensive and standardized definition of “low-risk” that incorporates a broader array of medical and obstetric factors to further refine risk stratification and enhance the generalizability of the findings.

Despite the limitations, the strengths of this analysis included a large number of deliveries from 18 monitoring hospitals of different geographic locations and grades, and the volume of data was generalizable. These findings (even with potential confounding) have potential implications for the management of low-risk women who labor at 37–41 weeks gestation, providing insight for women who meet eligibility criteria and clinicians planning trials, as well as contributing to cost-effectiveness analyses and health policies for low-risk pregnant women. Parity itself served as an easily accessible identifier for higher-risk populations. Clearly identifying these limitations paved the way for future, more rigorous studies designed to prospectively collect these variables.

## 5 Conclusion

To our knowledge the present study is the first to evaluate the differences in the distribution of pregnancy risk factors and adverse pregnancy outcomes between nulliparous women and multiparous women who labored at 37–41 weeks of gestation in China. Our findings suggests that the risk of maternal adverse outcomes and the incidence of complication interventions in nulliparous women are higher than in multiparous women. As a consequence, in addition to considering the gestational age for pregnant women, prenatal education, inspection and standardized management intervention should be carried out according to parity and gestational weeks of delivery, so as to reduce the occurrence of maternal and neonatal adverse outcomes.

## Data Availability

The original contributions presented in the study are included in the article/supplementary material, further inquiries can be directed to the corresponding author. The studies involving humans were approved by the Ethics Committee of Hunan Provincial Maternal and Child Health Care Hospital (2020S072). The studies were conducted in accordance with the local legislation and institutional requirements. The participants provided their written informed consent to participate in this study.
